# Real-Time Nanopore Q20+ Sequencing Enables Extremely Fast and Accurate Core Genome MLST Typing and Democratizes Access to High-Resolution Bacterial Pathogen Surveillance

**DOI:** 10.1128/jcm.01631-22

**Published:** 2023-03-29

**Authors:** Gabriel E. Wagner, Johanna Dabernig-Heinz, Michaela Lipp, Adriana Cabal, Jonathan Simantzik, Matthias Kohl, Martina Scheiber, Sabine Lichtenegger, Ralf Ehricht, Eva Leitner, Werner Ruppitsch, Ivo Steinmetz

**Affiliations:** a Diagnostic and Research Institute of Hygiene, Microbiology and Environmental Medicine, Medical University of Graz, Graz, Austria; b Austrian Agency for Health and Food Safety, Vienna, Austria; c Medical and Life Sciences Faculty, Furtwangen University, Villingen-Schwenningen, Germany; d InfectoGnostics Research Campus, Centre for Applied Research, Jena, Germany; e Leibniz-Institute of Photonic Technology (Leibniz-IPHT), Jena, Germany; f Friedrich Schiller University Jena, Institute of Physical Chemistry, Jena, Germany; Westfalische Wilhelms-Universitat Munster

**Keywords:** *Bordetella pertussis*, bacterial typing, molecular surveillance, next-generation sequencing

## Abstract

Next-generation whole-genome sequencing is essential for high-resolution surveillance of bacterial pathogens, for example, during outbreak investigations or for source tracking and escape variant analysis. However, current global sequencing and bioinformatic bottlenecks and a long time to result with standard technologies demand new approaches. In this study, we investigated whether novel nanopore Q20+ long-read chemistry enables standardized and easily accessible high-resolution typing combined with core genome multilocus sequence typing (cgMLST). We set high requirements for discriminatory power by using the slowly evolving bacterium Bordetella pertussis as a model pathogen. Our results show that the increased raw read accuracy enables the description of epidemiological scenarios and phylogenetic linkages at the level of gold-standard short reads. The same was true for our variant analysis of vaccine antigens, resistance genes, and virulence factors, demonstrating that nanopore sequencing is a legitimate competitor in the area of next-generation sequencing (NGS)-based high-resolution bacterial typing. Furthermore, we evaluated the parameters for the fastest possible analysis of the data. By combining the optimized processing pipeline with real-time basecalling, we established a workflow that allows for highly accurate and extremely fast high-resolution typing of bacterial pathogens while sequencing is still in progress. Along with advantages such as low costs and portability, the approach suggested here might democratize modern bacterial typing, enabling more efficient infection control globally.

## INTRODUCTION

The advent of next-generation sequencing (NGS) technologies led to a revolution in the field of medical diagnostics (1–3). Whether in human genetics, pathology, or medical microbiology, the high throughput and accuracy combined with decreasing costs have led to a plethora of new methods and approaches (4–7). It also represented a significant advance in molecular pathogen surveillance, as differences between isolates could be studied by whole-genome approaches at a nucleotide level (8–11). Due to not only the spread of new pathogens but also the emergence of new variants (e.g., host adaptation/escape mutations, antibiotic resistance), the study of genetic diversity and evolution of circulating strains is essential for the establishment and coordination of local and global countermeasures (8, 12). Here, NGS allows not only a detailed investigation of virulence genes (13, 14) and vaccine drift (15) but also high-resolution phylogenetic (14, 16, 17) and outbreak analyses (18, 19) at a previously unmatched level of detail.

Despite the importance and implications for our health care system, the widespread use of NGS technologies, essential for comprehensive pathogen surveillance, is still hindered by high acquisition costs and poor cost-efficiency, with low sample numbers and elaborate workflows (8, 20). This is further complicated by the complex analysis, which requires high computational power and bioinformatic skills (12, 20). Taken together, these represent a significant challenge for small- to medium-sized laboratories, especially in resource-limited settings. This is problematic in that on-site sequencing reduces the time to result significantly and thus allows faster intervention.

In the case of bacterial pathogens, core genome multilocus sequence typing (cgMLST) offers an elegant approach to addressing the bioinformatics bottleneck by standardizing and simplifying analyses. Typing isolates using a variety of conserved loci enables moderate computational requirements with high discriminatory power (21). Furthermore, the approach can easily be extended, allowing fast and easy screening for other genetic markers (e.g., resistance genes) and is, therefore, used routinely for a wide range of bacterial pathogens (18, 22, 23).

The missing sequencing capacity, in terms of available NGS instruments and sequencing output, was addressed by the wide use of nanopore sequencers and specifically optimized workflows during the recent SARS-CoV-2 pandemic (24–26). Nanopore sequencing offers the advantage of low acquisition costs, cost-efficiency, even in the case of low sample numbers, and a small footprint (USB stick-sized) compared to conventional short-read (SR) NGS technologies (1, 20). However, due to its inherent high error rate (1, 27), its application in molecular surveillance of bacterial pathogens has been limited in the past to analyses with lower resolution (28) or the profiling of microbial communities (29, 30). In the case of high-precision analyses, such as outbreak investigations of slowly evolving pathogens or the search for molecular markers based on single nucleotide polymorphisms, each additional incorrect base/allele difference might obscure the conclusions drawn (28). The cgMLST-based typing is especially sensitive to sequencing errors since it makes no difference whether one or hundreds of bases change, the difference between the respective alleles is always the same (equaling a different allele number). Although elegant workarounds that reduce these errors have been proposed, such as the *in silico* correction of reads in the case of Salmonella enteritidis (31), there is still a clear need for further improvements in routine high-resolution bacterial typing. With the continuous improvement of the software to minimize the impact of nanopore’s error rate (base caller, assembler, polisher [1, 32, 33]) and especially due to the recent release of Q20+ chemistry and flow cells (34), it is time to revisit the nanopore cgMLST-based molecular surveillance of bacterial pathogens.

Here, we applied the combination of nanopore sequencing and cgMLST analysis to Bordetella pertussis, the causative agent of whooping cough. Pertussis is a highly contagious but vaccine-preventable disease (35). Particularly (unvaccinated) infants and young children have a higher risk of severe disease progression (36), with reported case fatality rates of up to 7.2% for infants (37). There is a clear need for high-resolution molecular surveillance at a low threshold with the recent emergence of macrolide-resistant strains (38), antigenic drift (39), and the resurgence of pertussis observed generally (40). B. pertussis represents the ideal model bacterial pathogen for our evaluation because, as a very slowly evolving pathogen (41), particularly high demands must be imposed on the sequencing quality to account for minimal genetic differences. Due to its high infectivity (35), it is also a prime example where rapid typing plays a crucial role in containment and invoking adequate countermeasures.

We pursued two goals with our study, (i) to assess the potential of Q20+ nanopore sequencing for high-resolution bacterial pathogen typing (e.g., cgMLST, virulence factors, and resistance marker screening) by comparing its performance to gold-standard short-read technology, and (ii) to establish a workflow based on real-time sequencing data availability to achieve maximum speed for high-resolution bacterial typing.

## MATERIALS AND METHODS

### Isolation of genomic DNA.

Bordetella pertussis strains were cultivated on BD Bordet Gengou agar with 15% sheep blood (Becton, Dickinson) for 72 h at 37°C. After cultivation, DNA was isolated using the NucleoSpin microbial DNA kit (Macherey-Nagel) according to the manufacturer’s instructions, with slight modifications. (i) Samples were treated with RNase before application to the NucleoSpin microbial DNA columns. Therefore, after the incubation step at 70°C to inactivate proteinase K, 4 μL of 100 mg/mL RNase (Carl Roth) was added to the samples, which were then incubated for 5 min at 37°C. (ii) Genomic DNA was eluted in PCR-grade water (Roche) instead of elution buffer in the final step.

### Library preparation.

Genomic DNA was subjected to a magnetic bead clean-up before library preparation. Accordingly, DNA was mixed with an equal amount of AMPure XP bead solution (Beckman Coulter) and incubated for 5 min on a rotator mixer at room temperature. Afterward, beads were pelleted on a magnetic rack and washed twice with freshly prepared 70% ethanol, and genomic DNA was eluted with high-quality water by incubation for 10 min at room temperature.

DNA concentrations were determined using the Qubit BR assay kit (Thermo Fisher Scientific) on a Qubit 4 fluorometer (Thermo Fisher Scientific).

Libraries for nanopore sequencing were prepared using the native barcoding kit 24 (Q20+) (catalog no. SQK-NBD112.24; Oxford Nanopore) according to the manufacturer’s instructions.

### Nanopore sequencing, basecalling, and data preprocessing.

Prior to sequencing, a flow cell check was performed, and flow cells with less than the guaranteed 800 pores were replaced. The number of pores varied substantially, with even 1,200 pores not being uncommon. Since this also affects the sequencing speed and output (and hence, more coverage if the number of samples is fixed), the times given in the publication only serve as an estimate for the reader.

We sequenced 90 ng of the libraries on a MinION Mk1B (Oxford Nanopore) equipped with R10.4 flow cells (Oxford Nanopore), respectively. Pore occupancy (>70%) was monitored to ensure that a sufficient library was loaded. The minimal fragment length was set to 200 bp in MinKNOW. Data were basecalled and demultiplexed using Guppy 6.0.6 configured for R10.4 flow cell basecalling in superaccurate (SUP) mode. Subsequently, Duplex Tools 0.2.9 with default parameters was applied to all data sets for read splitting, as suggested by Oxford Nanopore. Seqtk 1.3 (42) was used to downsample sequencing data to the specified coverage denoted in the text.

### *De novo* assembly of long-read data.

All commands were run with default parameters if not stated otherwise in parentheses. Quality filtering was performed with NanoFilt (43) (q 10) to a quality score higher than 10. As specified in the text, the reads were assembled with the following *de novo* assemblers: Flye assembler v2.9 (44) (--nano-hq), miniasm v0.3 (45) and minipolish v0.1.2 (46), Raven v1.8.1 (47) (--graphical-fragment-assembly), and Canu v2.2 (48) (-fast genomeSize=4200000 stopOnLowCoverage=0 minInputCoverage=0 -nanopore-raw).

### Polishing of long-read assemblies.

Optional Racon polishing was carried out by mapping the filtered reads to the assembly with minimap2 v2.24 (49) (-ax map-ont). Assemblies were subsequently polished with Racon v1.3.1 (32) (-m 8 -x -6 -g -8 -w 500).

As suggested by Oxford Nanopore Technologies (ONT), Medaka polishing was always performed regardless of whether Racon polishing was applied or not. Therefore, all long-read (LR) assemblies were subjected to Medaka v1.5.0 (-m r104_e81_sup_g5015) polishing to obtain the final consensus assemblies.

In the case of hybrid assemblies, the final Medaka-polished Flye assemblies were refined with Illumina short reads. Therefore, Trimmomatic (50) v0.39 was used to filter the short reads (HEADCROP:10 SLIDINGWINDOW:5:20), and polishing was performed by Polypolish (51) (-v 0.6).

### Nanopore live basecalling run.

The live basecalling run was carried out using MinKNOW 22.05.5, with enabled SUP mode basecalling and enabled barcode demultiplexing. Sequencing coverage was monitored on barcode level, and the assembly pipeline (as described above) was triggered once an isolate reached the coverage thresholds of 20×, 30×, 40×, and 50× and finally once the run was finished.

We suggest reducing the number of reads per file from 4,000 to 1,000 in the Output Format panel of the MinKNOW graphical user interface (GUI) to benefit from even faster data availability in case of live basecalling.

### Short-read sequencing and assemblies.

Library preparation was performed using the Nextera XT DNA sample preparation kit (Illumina) followed by 300-bp paired-end whole-genome sequencing on an Illumina MiSeq, as described previously (52).

Short reads were assembled with SKESA 2.3.0 (53) using Ridom SeqSphere+ v7.8.0 (Ridom GmbH) and standard parameters.

### B. pertussis typing.

B. pertussis isolates were typed in Ridom SeqSphere+ v7.8.0 using our published cgMLST scheme containing 2,983 targets in the core genome and 179 targets in the accessory genome based on the vaccine strain Tohama I (GenBank accession no. NC_002929) as the seed genome (52). The scheme was chosen because it offers the highest resolution for cgMLST-based typing of Austrian B. pertussis isolates (52). The Illumina short-read data (newly acquired or from our previous study [52]) served as a reference for our analysis. Because only found targets can be evaluated for their sequence agreement, missing targets were not considered in the direct pairwise comparison for the comparison between different strains or sequencing technologies. Distance matrices were generated in SeqSphere+ by cross-comparison of the individual alleles of the found target genes and summation of the number of different alleles between isolates. Minimum spanning trees (MSTs) and neighbor-joining trees were created in SeqSphere+ using default parameters and the option “pairwise ignore missing values” in the case of missing targets. The cluster threshold was set to 6 in agreement with our previous publication. It is noteworthy that the SeqSphere+ missing values category includes “not found” and “failed” targets.

The BIGSdb-Pasteur *Bordetella* database (22, 54) was used to screen for vaccine antigens, virulence factors, and macrolide resistance genes with the following schemes: Bp_vaccine antigens, macrolide resistance, autotransporters, T3SS, phase, and other toxins. Genotype profiles (allele combinations) were assigned based on allele query results for the vaccine antigens (Table 1). In the case of the *prn* locus, several alleles had not yet been assigned. In this case, the genotype was determined by matching the respective extracted sequences.

**TABLE 1:** Definition of genotype profiles based on the allele assignment for the loci denoted by the online *Bordetella* database (22, 54)

Genotype profile	*ptx*S1^*a*^	*ptx*P	*fim*3	PRN-Bp	*fim*2	Corresponding genotype in previous study (52)
A	A^*b*^	3	1	2	1	*ptx*S1-A/*ptx*P-3/*prn*-2/*fim*2-1/*fim*3-1
B	A	3	1	27	1	*ptx*S1-A/*ptx*P-3/*prn*-2-631^632STOP: T>-/*fim*2-1/*fim*3-1
C	A	3	2	2	1	*ptx*S1-A/*ptx*P-3/*prn*-2/*fim*2-1/*fim*3-2
D	A	3	1	D^*c*^	1	*ptx*S1-A/*ptx*P-3/*prn*-2-303+1326DEL/*fim*2-1/*fim*3-1
E	A	3	2	E^*c*^	1	*ptx*S1-A/*ptx*P-3/*prn*-2-IS481-1613rev/*fim*2-1/*fim*3-2
F	A	3	1	F^*c*^	1	*ptx*S1-A/*ptx*P-3/*prn*-2-IS481-1613rev/*fim*2-1/*fim*3-1
H	A	3	1	22	1	*ptx*S1-A/*ptx*P-3/*prn*-2-T223C/*fim*2-1/*fim*3-1
I	A	3	1	24	1	*ptx*S1-A/*ptx*P-3/*prn*-2-C1273T/*fim*2-1/*fim*3-1
X	A	3	1	X^*c*^	1	Genotype not present in our previous study; *ptx*S1-A/*ptx*P-3/*prn*-2-IS481-2180fwd/*fim*2-1/*fim*3-1

a *ptx*S1 represents an allele combination of six different loci (*ptx*-A, *ptx*-B, *ptx*-C, *ptx*-D, *ptx*-E, and *fhaB*).

b Genotype A alleles include 1, 1, 4, 1, 4, and 1.

c Alleles were found but had not yet been deposited in the database.

### B.NanoAmP, a bacterial nanopore assembly pipeline.

We developed a Python-based tool with a GUI called B.NanoAmP to simplify the creation of a customized pipeline (selection of tools and adjustment of settings). It is freely available (https://github.com/simanjo/B.NanoAmP) and comes with predefined, downloadable conda environments for easy, automated installation of the pipeline tools, which requires only an existing conda installation. In principle, it works as a wrapper for the assembly tools in order to modify their settings easily and, as such, can be used for the assembly generation and its polishing of any bacterial species. The pipeline includes Filtlong v0.2.1 (55) read filtering by supplying the genome size and a threshold for minimal read length. Furthermore, the user can choose between Flye, Raven, and miniasm assembler (or use all simultaneously) and polishing procedures (Medaka with or without Racon). In order to employ the full potential of Medaka, the model used in the polishing step can be chosen either from the full list of models available or by interactively selecting the important experimental features (pore type, device, and guppy version used for basecalling) followed by an educated choice of the best-fitting configuration. Once executed, the tool will process the files in the folder selected containing either reads or subfolders containing reads, where each subfolder is interpreted as a single assembly task originating, for example, from different barcodes.

### Statistical analysis and data visualization.

Statistical analysis was carried out in R version 3.6.3 using RStudio version 2022.02.0+443 (RStudio, Inc.). Quade test and Wilcoxon signed rank *post hoc* testing were used to test significance of missing targets in assemblies of different assemblers. In *post hoc* testing, the *P* values were adjusted for multiple testing by the method of Holm.

Figures were created with ggplot2 in RStudio (RStudio, Inc.), BioRender (https://www.biorender.com/), SeqSphere+ (Ridom GmbH), and/or FigTree 1.4.4 (56).

### Data availability.

Newly sequenced raw reads have been deposited under BioProject accession no. PRJNA853901 in the National Center for Biotechnology Information Sequence Read Archive repository. The corresponding sequencing data are listed in Table S1 in the supplemental material.

Illumina short-read data from our study published previously (38) was used (where data were deposited under BioProject accession no. PRJNA642701) for comparison to our LR assemblies.

## RESULTS AND DISCUSSION

### Establishment of a robust pipeline for Q20+ nanopore sequencing and data processing for subsequent cgMLST analysis.

At present, it is still unclear whether nanopore sequencing data can reach the required accuracy needed for the high-resolution typing of bacterial pathogens. Our own attempts to conduct B. pertussis cgMLST-based analysis using the previous chemistry and generation of flow cells showed that nanopore sequencing was not yet an alternative for highly accurate bacterial typing (see Fig. S1 in the supplemental material). An error profile resulting in allelic differences up to six is clearly too high, especially considering that even epidemiologically unrelated strains were reported to be nearly indistinguishable for B. pertussis (54) and even more considering the published cluster threshold of six of the cgMLST scheme applied here (52). Craddock and colleagues made similar observations for Brucella melitensis (28). Although a recently published study by Sereika and colleagues (34) demonstrated a large leap in the quality of bacterial assemblies using ONT’s novel Q20+ chemistry, the eligibility for cgMLST analysis remains to be determined. Especially due to the real-time availability of the data and the associated possibility of analyzing a small number of samples within 1 working day, nanopore sequencing would be an extremely valuable tool in the field of molecular epidemiology.

**FIG 1:**
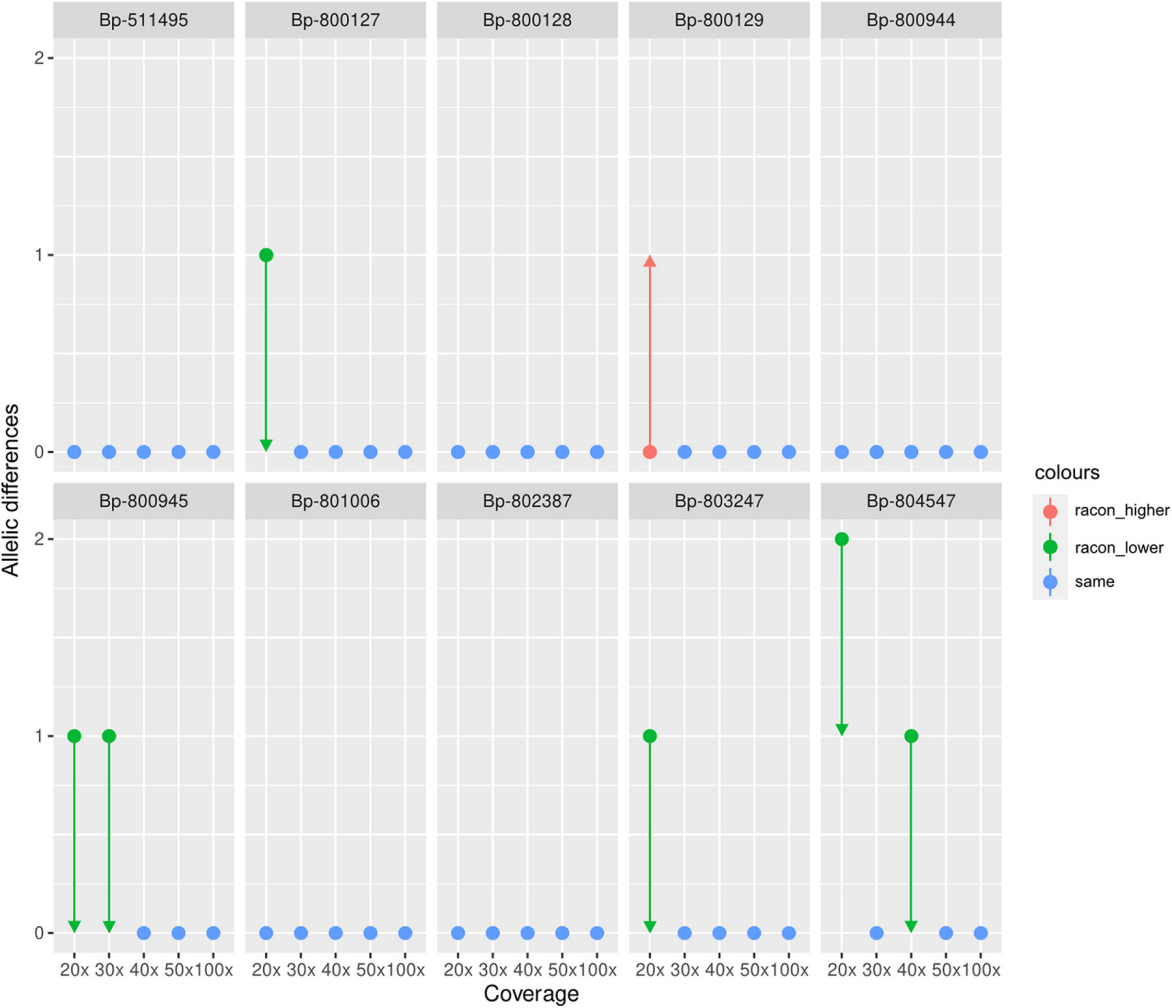
B. pertussis cgMLST (2983 loci) allelic differences between the Q20+ LR-AS and gold-standard SR-AS serving as a reference. Racon polishing was evaluated as an optional step in addition to Medaka polishing. Green arrows represent a decrease and red arrows an increase in the allelic differences if optional Racon polishing was applied.

We therefore used a subset of 10 B. pertussis strains (chosen strains are denoted in Fig. 1) to evaluate the novel Q20+ chemistry for cgMLST analysis and refine a pipeline to ensure fast processing, high-quality assemblies, and robust subsequent analysis. As our goal was to use the final assemblies for gene-by-gene approaches due to the ease of use, computational demands, and scalability, cgMLST allelic profiles served as a readout, and SKESA short-read assemblies (SR-AS) were used for comparison. Strains were selected to include closely and distantly related strains alike, covering allelic distances between different strains from 0 to 19 in the SR-AS. First, we assessed the impact of the LR assembler on the cgMLST profile. The assemblers Flye, Raven, miniasm/Minipolish, and Canu were chosen based on a detailed comparison by Wick and colleagues (46), where they were shown to offer highly accurate assemblies. Medaka polishing was performed, regardless of the assembler applied, to obtain the final consensus LR assemblies (LR-AS), as suggested by ONT.

We carried out an assessment of the allelic profiles of all the assemblies (4 LR-AS plus reference SR-AS per strain) from every single isolate. Indeed, the novel chemistry resulted in genome assemblies that were almost indistinguishable from the SR data of the respective strain. In the case of the respective alleles present of all LR-AS, agreement was 100% with the SR-AS for all isolates except for the miniasm assembler. The latter differed in one isolate (Bp-803247) from all other assemblies in one allele. In order to analyze the discrepancy, we polished the miniasm assembly with SR data. Indeed, there was no difference between the identified alleles of the hybrid assembly and the other ones, confirming that the allele in the initial miniasm assembly was wrong. No differences were found in the present accessory genome alleles of any of the assemblies of the 10 strains, respectively.

These observations are nicely reflected in the distance matrix based on a pairwise comparison of all assemblies of the 10 strains (Tables S2 and S3). Numbers represent the allelic distance between the isolates; therefore, blocks of zeroes at the diagonal can be used to infer agreement between different assemblies of the same isolate.

Of note, although a GC bias has been reported for nanopore sequencing (27), it was not an issue for our cgMLST analysis of B. pertussis with its rather high GC content of ~67%.

It is clear that the correct allele assignment is the essential factor of cgMLST typing; however, the number of targets found is similarly a crucial parameter. The latter contributes directly to the resolution of a comparison, as it makes a difference how many targets are available for differentiation between the strains. As can be seen in Table 2, the choice of the assembler directly affected the average number of missing targets in the respective LR-AS, which were significantly reduced compared to SKESA SR-AS.

**TABLE 2:** Comparison of missing targets for different assemblers of genomic data of 10 strains (*n* = 2,983 loci)

Assembly	Avg no. of contigs	Median no. (IQR)^*b*^ of missing cgMLST targets
LR Flye + SR polishing	1.0	8.0 (7.0–11.0)
LR Flye	1.0	8.0 (8.0–12.0)
LR miniasm/Minipolish	1.0	9.0 (9.0–12.75)
LR Raven	1.0	9.0 (9.0–14.0)
SR SKESA^*a*^	365.5	174.0 (162.25–180.75)
LR Canu^*a*^	21	368.0 (274.25–438.5)

a Significantly different from every other assembler.

b IQR, interquartile range.

A statistically significant difference was found between the missing targets of the Canu assembler and every other assembler (adjusted *P* ≤ 0.011 in all cases), and the same was found for SKESA (adjusted *P* ≤ 0.011 in all cases). A detailed comparison, including adjusted *P* values, can be found in Table S4.

The low number of missing targets is probably a result of the contiguity of the assemblies (see the average number of contigs in Table 2).

In summary, we provide evidence that the LR-AS of Flye and Raven are highly accurate and, therefore, indeed suitable for cgMLST-based analysis. The assemblies are characterized by a low number of missing targets, and the allelic variants identified matched those of the gold-standard SR-AS.

The Flye assembler was chosen for our subsequent detailed analysis because it tends to be better regarding missing values and is the assembler recommended by ONT. Nevertheless, Raven and, with restriction, miniasm/Minipolish could also be used. Compared to Flye, the runtime is reduced (46), which can prove to be an advantage in the case of older hardware. However, since we found an incorrect allele in a miniasm assembly, the assembler should be evaluated for the application of choice before use. Canu is not a real alternative because its LR-AS have a drastically increased number of missing targets, and the runtime is also considerably higher than that of other assemblers (46).

We next investigated the impact of the sequencing coverage and optional Racon polishing (in addition to Medaka) on the cgMLST-typing performance.

Figure 1 indicates that the optimal assemblies for cgMLST typing were obtained at a coverage of 50× when only Medaka polishing was performed, but with certain differences between the strains below that value.

The addition of Racon polishing further improves the assemblies, as, down to a coverage of 30×, no differences to the SR-AS were observed. Below that value, Racon polishing still seems beneficial except for one single strain (Bp-800129). Therefore, we suggest that careful consideration should be taken when analyzing data at or below this coverage.

Since our goal was to provide a user-friendly high-resolution molecular surveillance of pathogens, we incorporated these observations into the development of an assembly pipeline with a GUI (see Fig. S3). Our bacterial nanopore assembly pipeline (for short, B.NanoAmP) should simplify the entry into cgMLST-based analysis for users with limited bioinformatics knowledge, facilitating and automating the assembly generation for subsequent analysis with available tools such as SeqSphere+ or chewBBACA-chewie-NS (57, 58). The tool is freely available, and conda environments are provided for easy installation. It may serve as a general assembly and polishing interface for all kinds of bacterial species and is not limited to B. pertussis.

### Low-threshold nanopore sequencing catches up with gold-standard short-read technologies for high-resolution bacterial pathogen typing.

**FIG 2:**
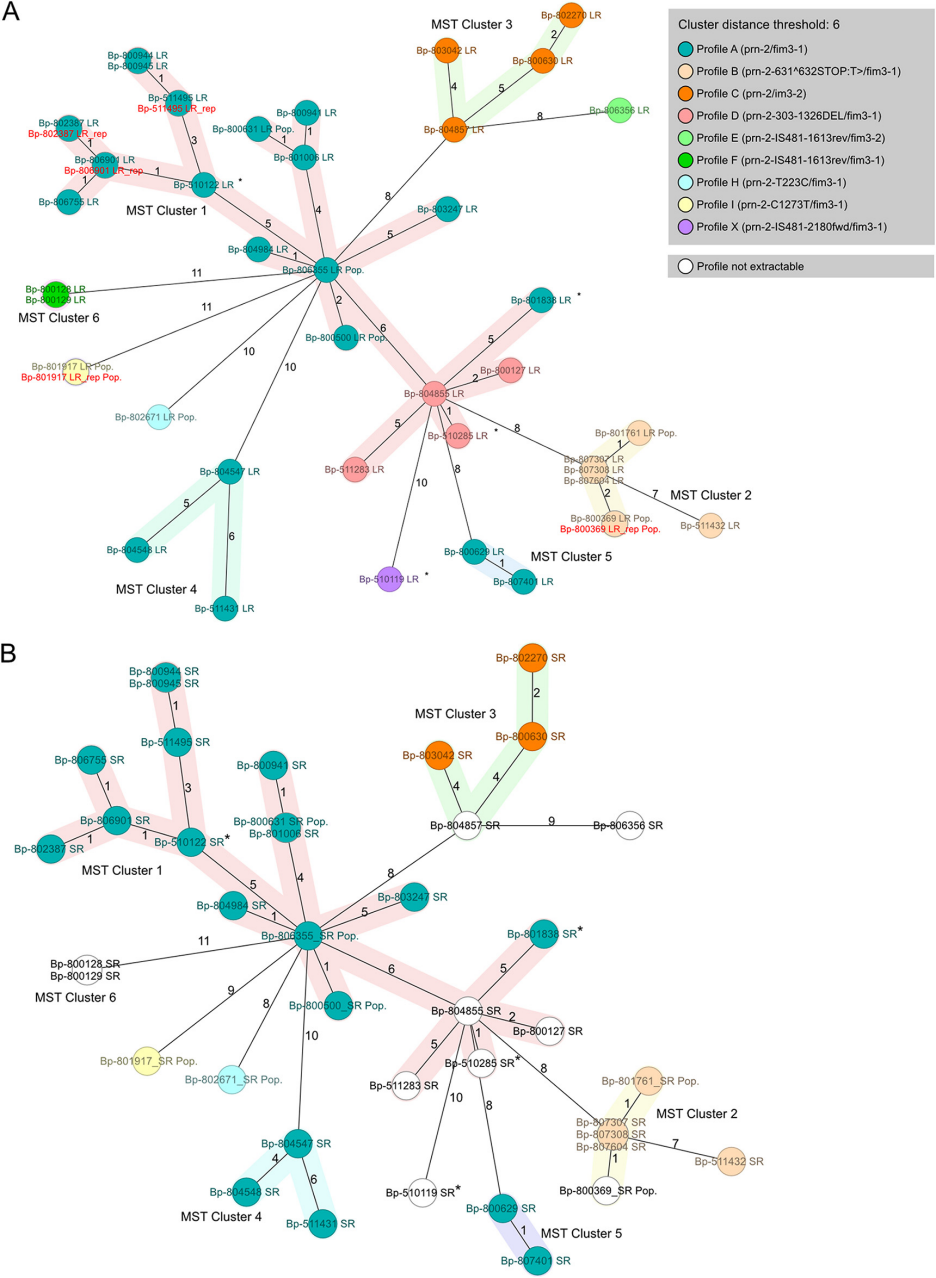
Comparison of cgMLST-based minimum spanning trees (MSTs) of LR-AS (A) and reference SR-AS (B), each comprising the same 40 B. pertussis strains, shows the great performance of LR-AS for high-resolution molecular typing. The respective clusters are preserved independent of the sequencing technology used. The same is observed for allelic differences between isolates: minimal differences between the trees are not a result of sequencing errors but can be attributed to differences in the number of missing targets and one base ambiguity. Numbers on lines indicate the number of allelic differences between the respective strains. The cluster threshold was set to six. Replicates are indicated by the red font color. *, new strains; bacterial populations contain “Pop.” Nodes/strains are colored by genetic profile, whereas only differences in the genotype are denoted (Loci *ptx*S1-A, *ptx*P-3, and *fim*2-1 are conserved in the strains presented here).

These promising results led us to investigate whether our nanopore Q20+ chemistry-based workflow is suitable for the broadly applicable molecular surveillance of bacterial pathogens. We aimed for at least 100× coverage to accommodate for potential variations from the preliminary results shown above (hence, there was also no need for additional Racon polishing). In our experience, the typical yield of the new flow cells is at least 10 Gb, so even in the case of 100× coverage, this estimates sequencing 20 B. pertussis strains. We made use of 30 closely and distantly related Austrian B. pertussis strains from our previous study (52) to investigate its potential for high-resolution outbreak analysis. The collection was supplemented with four new strains and seven bacterial populations. The term “bacterial population” is in concordance with a previous B. pertussis study in which sequencing revealed a nucleotide mixture in a certain locus (59), indicating the possible existence of more than one strain in a certain sample. While the biological relevance is out of the scope of this work, we wanted to address the question whether or not Illumina and nanopore sequencing show comparable results in such a case. Our comparison relied on MSTs for analysis, as they are extensively used in outbreak investigations facilitating the identification of clusters and transmission events. As can be seen in Fig. 2, the two MSTs of LR-AS (Fig. 2A) and SR-AS (Fig. 2B) not only showed the same clusters of isolates but also that the distances between isolates differed only minimally.

If distance differences between the two trees were observed, they were actually due to additionally found and correctly identified alleles, as a comparison with the tree of hybrid assemblies (HYB-AS) shows (Fig. S4). Only the distance between strains Bp-804857 and Bp-806356 was increased by one in SR MST, which was due to the unidentified allele of locus BP0986 in Bp-804857 LR-AS.

Even in the case of the bacterial population samples, the typing was consistent for 6/7 strains. There was a single difference here in the case of Bp-800631. The allele in locus BP0985 differed in the assemblies (LR-AS versus SR-AS), which is exactly the locus and the nucleotide differing in the bacterial population sample. If reads were mapped to the assemblies, reads of both technologies clearly showed the base ambiguity at this position (see Table S5). This implies that (i) even in the case of bacterial populations, similar typing results are likely to be observed regardless of whether SR-AS or LR-AS are used, and (ii) in the case of divergent results, one should check for bacterial populations by means of read mappings, as it is not automatically an indication of a sequencing error.

Apart from the single exception due to the true base ambiguity in the population sample, the allele variants of the cgMLST typing identified are identical between all assemblies of the same strain. Additional description and discussion of the differences between the respective assemblies can be found in Tables S6 to S8, including distance matrices for an easy overview of the comparisons.

However, we would like to point out a potential advantage of LR-AS. In our previous study, the assignment of the *prn* genotype, which is of interest due to its relevance for vaccines (60), was only possible by mapping the short reads to the reference and subsequent visual inspection in the case of several pertactin variants. Our LR-AS allowed an easier identification of matching PRN types. Final assemblies were screened for the PRN target using the Institut Pasteur *Bordetella* database (22, 54) and tools (61). In the case of known allele variants, these could be identified and assigned directly; the PRN-Bp hit was provided for the others. The sequence of this hit is identical for isolates with the same *prn* genotype, which means that they can be matched easily. As shown in Fig. 2A, each strain in the LR-AS could be assigned a genotype (indicated by color). This is not the case for the SR-AS (Fig. 2B), where the inconsistent identification of the PRN target prevents the assignment of the genotype (shown in white) for several strains. The same might be true for problematic/long targets in other species.

**FIG 3:**
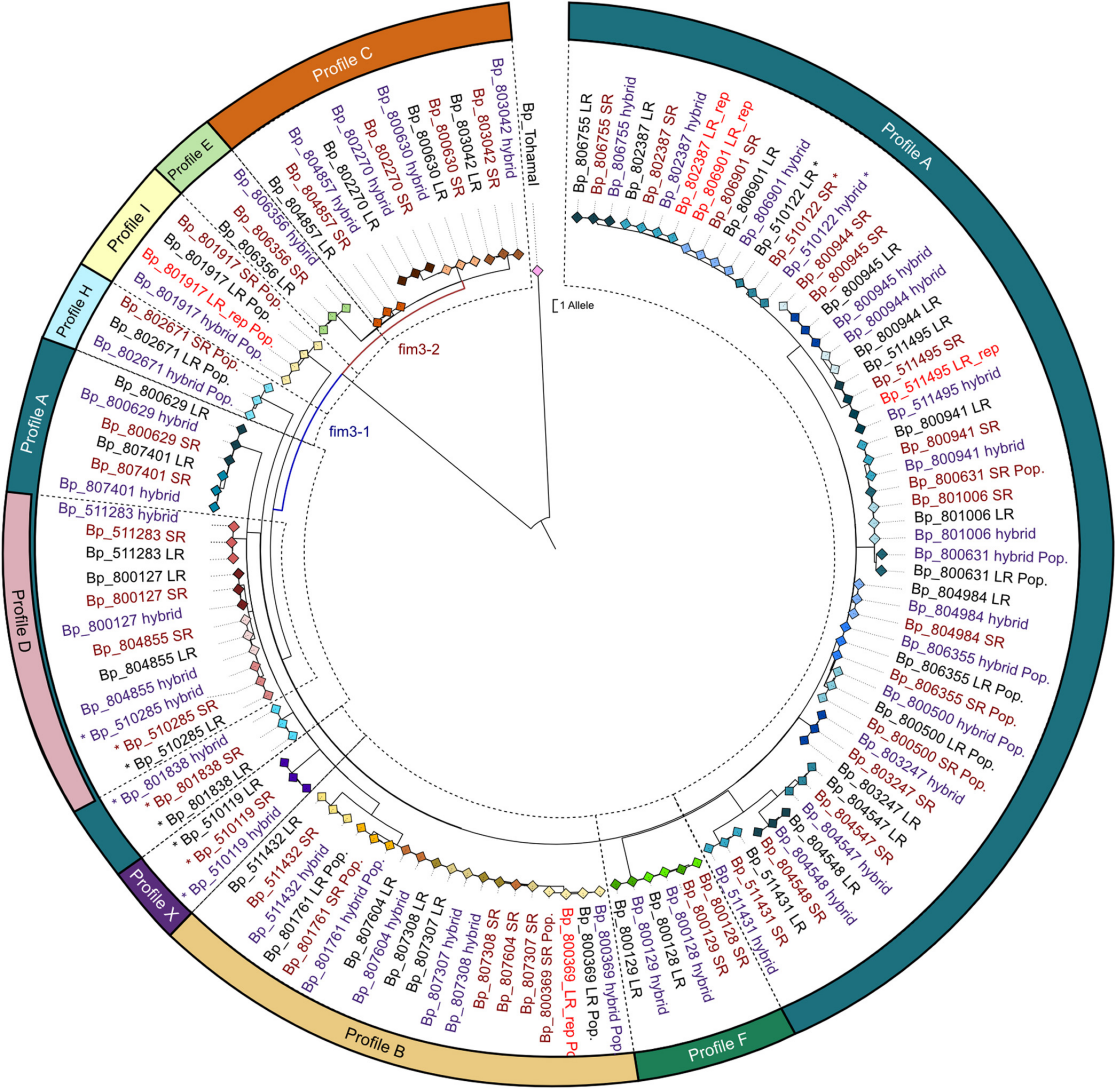
Neighbor-joining tree of 40 B. pertussis strains shows the high quality of nanopore assemblies, which cluster directly with their short-read pendants and the polished hybrid assemblies. The tree nicely reflects the B. pertussis phylogeny of Austrian strains (52) and underlines the applicability of nanopore data for cgMLST-based phylogenetic analysis. The tree was rooted in the vaccine strain Tohama I. Strains can be distinguished by the color of the symbols. LR-AS are indicated in black, SR-AS in dark red, and HYB-AS in a violet font color. Technical replicates are marked in a red font color and bacterial populations by “Pop.”

Considering these excellent results, it is no surprise that LR-AS are also suitable for phylogenetic analyses. The different assemblies of a given isolate cluster directly next to each other in a neighbor-joining tree (Fig. 3). Isolates can be divided into two clades based on *fim*3 (*fim*3-1 and *fim*3-2), whereas isolates with the same genetic profile cluster closely in further subgroups, except for profile A isolates. This is in agreement with our previous analysis of short-read data (52), and readers are referred to the aforementioned publication for an in-depth discussion.

Finally, we examined the robustness of nanopore sequencing and subsequent cgMLST typing. The LR-AS of the repeated nanopore sequencing of five strains lie exactly on/beside their replicates in the respective tree (Fig. 2A and Fig. 3, marked by the red font color), demonstrating its reproducibility.

Bacterial assemblies are generally useful for addressing a plethora of scientific questions. Bridel and colleagues have recently established a publicly available *Bordetella* database with an easy-to-use web interface that allows one to analyze macrolide resistance, virulence genes, and targets of acellular pertussis vaccines and make easily accessible cgMLST analyses *inter alia* (22). As before, excellent agreement between the results of the two sequencing technologies was observed when we screened our assemblies with several predefined schemes (e.g., vaccine antigens, macrolide resistance); see Table S9. As expected, nanopore sequencing is advantageous in the case of long targets, as can be seen in Table S9 in the case of the *fhaB* gene (1 to 10,773 bp). All vaccine targets were correctly identified, as shown by comparison with HYB-AS, demonstrating that the nanopore assemblies are also suitable for the analysis of vaccine escape variants. Likewise, the same results were obtained for the markers of macrolide resistance (all isolates classified as sensitive) and the analysis of virulence factors and toxins, except for a single challenging nanopore target (autotransporter locus tcfA). The incorrect allele in some of the LR-AS results from a homopolymeric stretch (10 consecutive Gs in allele 2 versus 9 Gs in allele 5). These regions are indeed problematic for nanopore technology (27, 62), and our HYB-AS proved that typing of the SR-AS was correct. While this is certainly not ideal, one also has to mention that this target was also not identified in 70% of the SR-AS (see Table S9). Furthermore, long homopolymeric regions are rather rare in bacteria, as shown by Sereika et al., analyzing more than 1,500 different genera, which is why they proposed that this should not be a huge issue in general (34). To put this into context, even in our study, it is only a problem of a single analyzed coding region out of over 3,000.

### Nanopore-based real-time molecular typing for bacterial pathogen surveillance.

**FIG 4:**
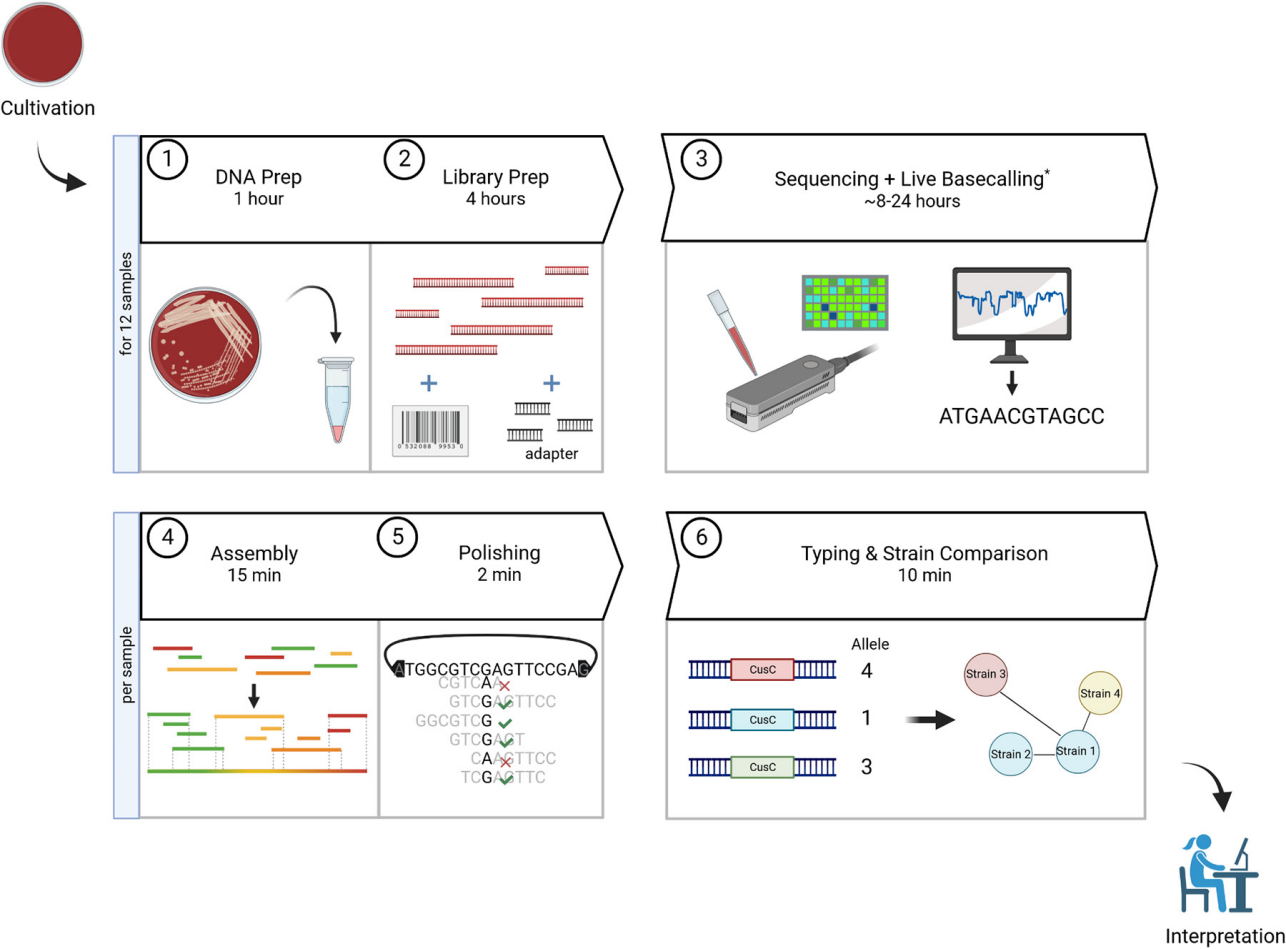
Q20+ nanopore sequencing cgMLST workflow. The time of each step is given for the number of samples denoted. Live basecalling on a dedicated GPU was used to circumvent any additional time-consuming postrun basecalling, which is absolutely essential for fast analysis. The duration of all other bioinformatic steps are rough estimates for standard desktop PCs (e.g., 8-Core CPU, 16 GB RAM, SSD drive). *, sequencing time varies greatly depending on the number of samples, genome size of species under investigation, the sequencing coverage desired, and also the pore count of the flow cell (see Materials and Methods for details). The time given is an estimate for 12 B. pertussis strains in our study to obtain 30 to 100× coverage (see paragraph on real-time molecular typing for detailed information regarding sequencing time and coverage). Created with BioRender.com.

Having demonstrated the suitability of nanopore sequencing for high-resolution molecular surveillance, we next wanted to exploit its feature of real-time data acquisition for the fastest possible bacterial typing. Our suggested workflow, including the time needed for the individual steps, is depicted in Fig. 4. We make use of a consumer-grade graphical processing unit (GPU), a GeForce RTX 3080 12 GB in our case, to use the time efficiently during sequencing to eliminate time-consuming postrun basecalling. It is noteworthy that a powerful GPU is absolutely essential to enable live basecalling in superior (SUP) mode and, thus, the rapid genomic characterization and analysis of isolate relationships at the highest possible quality. Besides this particular component, the rest of the analyses can easily be run on standard desktop hardware. Commercial kits and lab equipment, which should be readily available in diagnostic labs, are used.

Making use of a performant, dedicated GPU for live basecalling, sequence data are practically immediately available. This is an outstanding feature compared to sequencing by synthesis technologies, in which case the entire run must be finished so that the reads reach the defined read length. As such, it opens up new opportunities for rapid molecular surveillance. One can start to analyze the data as soon as a suitable coverage is reached, still acquiring additional reads for subsequent cycles of analysis.

In our approach, we start the sequencing run and live basecalling, monitor the data acquisition on a barcode/strain level, and start to process and analyze the data once certain coverage thresholds have been reached by a strain. Based on our downsampling analysis (Fig. 1), a first analysis was carried out at 20× coverage with subsequent rounds after each additional 10× up to 50× and a final evaluation at 100×. Sequencing was stopped once every sequenced isolate reached 100× coverage after 20.7 h. We chose a set of 10 strains, considering strains that cluster both near and far from each other, to investigate the impact on various distances between strains.

**FIG 5:**
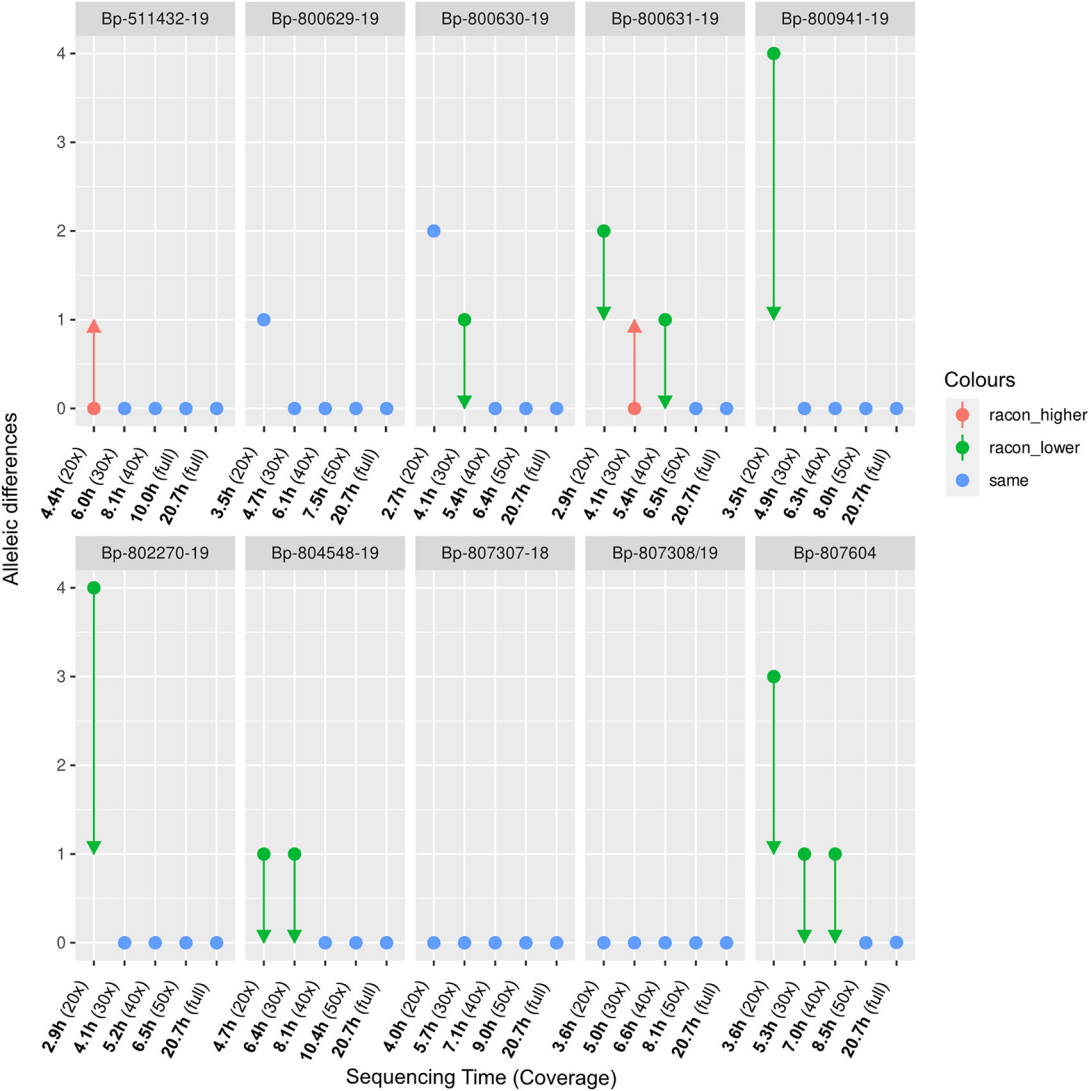
B. pertussis cgMLST (2,983 loci) allelic differences between the assemblies of live sequencing data at the time denoted (to achieve a certain coverage) and gold-standard SR-AS serving as a reference. Racon polishing was evaluated as an optional step in addition to Medaka polishing. Green arrows represent a decrease, and red arrows an increase in the allele differences if additional Racon polishing was applied.

The experimental data are in agreement with our downsampling analysis; the highest allelic differences between the assemblies and SR-AS reference are observed at 20× and decrease subsequently with increasing coverage (Fig. 5). Although no longer officially included by ONT, the addition of Racon polishing benefits the LR-AS at low coverages except for two strains at low coverage (Bp-511432-19 at 20× and Bp-800631-19 at 30×). Therefore, we suggest including Racon polishing for sequencing at lower coverages. Most of the time, it improves the assemblies, and if not, the introduced error is minimal (maximum of one incorrect allele). Using this approach, allelic differences of a maximum of two compared to the SR-AS reference were observed at the lowest coverage, in contrast to up to four if Racon polishing was not applied. At higher coverage, the error was further reduced to a maximum of one allele (Fig. 5).

**FIG 6:**
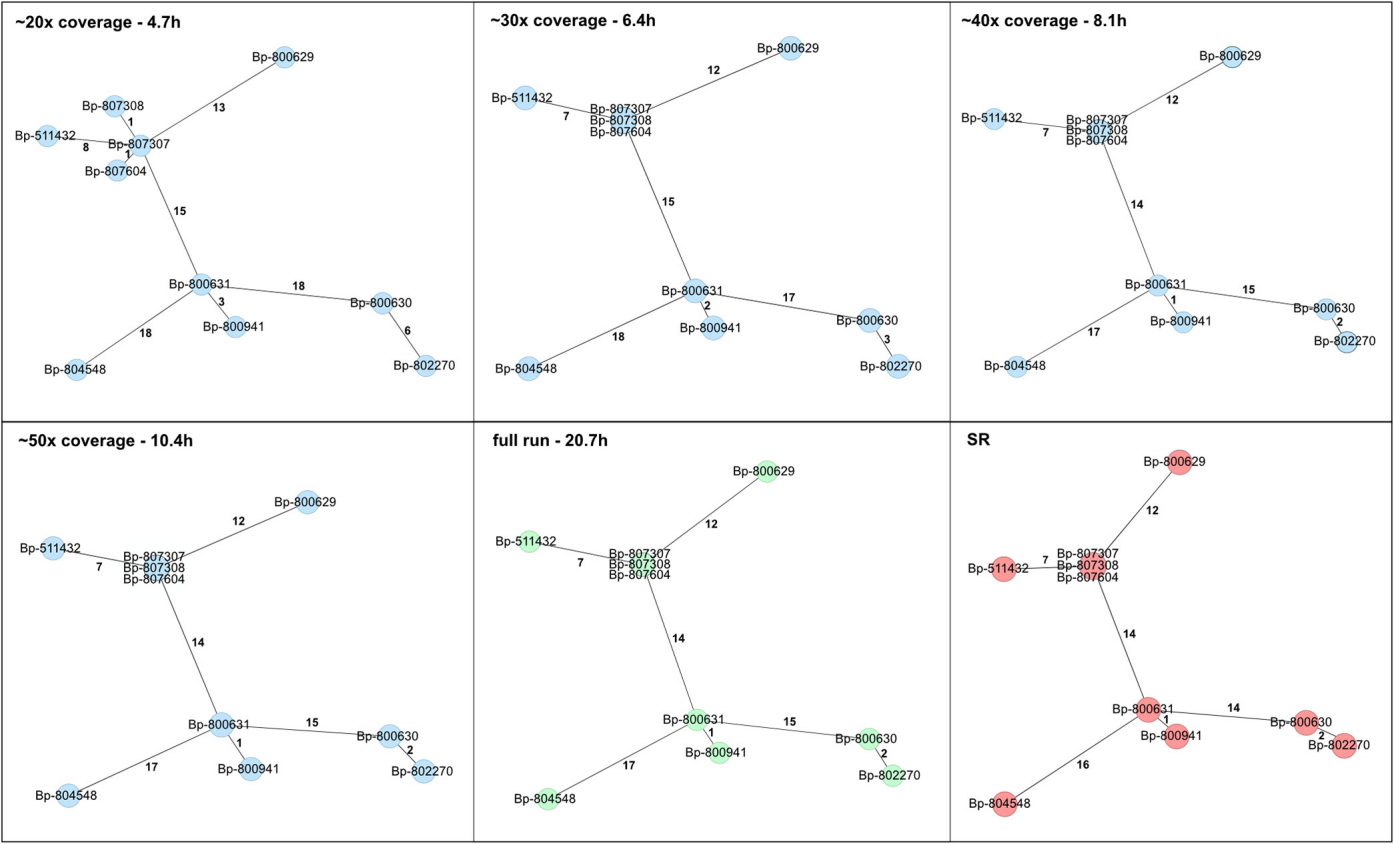
MSTs generated from a live basecalling run and cyclic analysis. The preliminary LR-AS trees are shown in blue, the final LR-AS tree (at least 100× coverage for all isolates) in green, and an SR-AS tree in red as reference. Although the tree at 20× coverage still has several errors, close isolates already indicate potential transmissions demanding further investigations based on epidemiological data. The 30× MST almost resembles the final one. No differences were observed between the other LR-AS-based trees. The differences from the SR-AS MST result from differences in missing targets and are not due to incorrect allele assignment. In agreement with our processing suggestion in the main text, optional Racon polishing (blue trees) was performed for assemblies of coverage of ≤50. The indicated time corresponds to the time at which the last sample reaches the respective coverage.

The benefits of our approach are well illustrated by the following MSTs resulting from the respective coverage (Fig. 6). The structure of the tree is clearly visible at 20×, but with significant errors in the isolate distances. Although one would definitely not exclude any isolate from an outbreak at this time, close isolates will already be attracting attention. This results from the fact that distances, if they change at all, are further reduced and not very likely to be increased at a higher coverage. The tree at 30× coverage almost resembles the final one; the maximum difference between the isolate distances of the two trees is already reduced to 1, thus enabling a close to a final evaluation of isolate relationships. No differences were observed for the trees at 40× and 50× coverage from the final evaluation. There were subtle differences from the SR-AS tree, which, again, can be traced back to the differences in number of missing targets but not incorrect alleles.

It is worth mentioning that even when repeating SR sequencing of an isolate, there may be minimal changes in its allelic profile and, thus, altered distances in, for example, MSTs. In addition, the cluster threshold is a rather soft classification criterion. In any case, isolates should never be assigned to an outbreak only on its basis; ultimately, additional epidemiological data are decisive for the assignment. From this point of view, the differences observed at low coverage are less problematic than one might think. However, this comes with the benefit of a much shorter time until one can start with the analysis. In the case of our set of 10 isolates on one flow cell, 20× coverage is reached between 2.7 and 4.7 h, depending on the strain (see Fig. 5). Afterward, it takes an average of 1.9 h for each additional 10× to reach 50× coverage.

Importantly, there is also a different mode of evaluation. There will be differences in the sequencing output between isolates and barcodes, even with state-of-the-art DNA quantification and accurate library prep. Therefore, some isolates reach the respective coverage earlier than others (see Fig. 5). This opens up interesting possibilities, as, frequently, not only the relationships of the isolates within a run are of interest but also those with deposited, sequenced strains in order to detect and trace transmission chains. Such a comparison is easily feasible by assembling and analyzing the respective genomes as soon as the appropriate coverage is met (e.g., after 2.1 and 4.7 h in the case of 20× and 30×, respectively, for the fastest isolates).

A common 2 × 150-bp sequencing run on an Illumina MiSeq, the mainstream instrument in small- to medium-sized diagnostic labs, takes approximately 24 h without library prep, according to the manufacturer (63). Either way, its high purchase price is a limitation for on-site application in small laboratories and low-resource settings. Taking into account 4 h of library prep, our approach allows a very detailed analysis of 10 isolates at 30× coverage within 8 to 10 h, i.e., 1 working day. Depending on the circumstances, increased cost can be traded for higher speed and vice versa in our approach; for example, the thresholds are theoretically reached 10 times faster when sequencing a single isolate (e.g., 30× after 30 min). While the potential costs for sequencing a single or only very few isolates might seem high, there is no doubt that the speed advantage in time to result will be highly cost-effective in many settings. Apart from ethical issues, timely pathogen typing resulting in hygienic measures, which can prevent, for example, nosocomial transmissions and potential closure of wards, will save costs that outweigh, by far, laboratory consumables.

### Conclusion.

We could show here that nanopore sequencing proved to be a reproducible, powerful, and competitive tool for the molecular surveillance of bacterial pathogens. Taking advantage of the recent advances in Q20+ chemistry, it enables the in-depth genetic analysis of consensus assemblies at the level of gold-standard short-read technology. We showed in our study that the LR-AS are ideally suited for easily applicable, standardized cgMLST-based analyses to explore isolate relationships. For this reason alone, nanopore sequencing has the potential to widen molecular surveillance and take our understanding of circulating strains to a whole new level. Furthermore, real-time data availability enabled the implementation of extremely fast data availability and analysis, which allows one to set, for example, hygienic countermeasures at an unprecedented speed.

This approach, combining nanopore sequencing and cgMLST analysis, offers several beneficial properties, including a low acquisition cost, a simple workflow, and standardized and streamlined analysis of the data, with minimal requirements of computational power and bioinformatics skills. Consequently, it provides an excellent foundation to democratize the molecular surveillance of bacterial pathogens and improve infection control measures in the future.
