# Supplementary material for: Real-Time Nanopore Q20+ Sequencing Enables Extremely Fast and Accurate Core Genome MLST Typing and Democratizes Access to High-Resolution Bacterial Pathogen Surveillance
Source: J Clin Microbiol. 2023 Mar 29;61(4):e01631-22. doi: 10.1128/jcm.01631-22 (PMC10117118; doi:10.1128/jcm.01631-22)
Supplement: Supplemental file 1 — Supplemental material. Download jcm.01631-22-s0001.pdf, PDF file, 1.7 MB [file jcm.01631-22-s0001.pdf]

Supplementary Real-time Nanopore Q20+ Sequencing enables extremely fast and accurate core genome MLST typing and democratizes access to high-resolution bacterial pathogen surveillance

Running title: Nanopore-based high-resolution pathogen surveillance

Gabriel E. Wagner[1*], Johanna Dabernig-Heinz[1#], Michaela Lipp[1#], Adriana Cabal[2], Jonathan Simantzik[3], Matthias Kohl[3], Martina Scheiber[1], Sabine Lichtenegger[1], Ralf Ehricht[4,5,6], Eva Leitner[1], Werner Ruppitsch[2], Ivo Steinmetz[1*]

[1] Diagnostic and Research Institute of Hygiene, Microbiology and Environmental Medicine, Medical University of Graz, Graz, Austria.

[2] Austrian Agency for Health and Food Safety, Währingerstraße 25a, 1096 Vienna, Austria

[3] Medical and Life Sciences Faculty, Furtwangen University, 78054 Villingen-Schwenningen, Germany

[4] InfectoGnostics Research Campus, Centre for Applied Research, Jena, Germany

[5] Leibniz-Institute of Photonic Technology (Leibniz-IPHT), Jena, Germany

[6] Friedrich Schiller University Jena, Institute of Physical Chemistry, Jena, Germany

[#]These authors contributed equally to this work

*Corresponding authors:

gabriel.wagner-lichtenegger@medunigraz.at and ivo.steinmetz@medunigraz.at

Material and methods

Data availability

Newly generated sequencing data were deposited in the Sequence Read Archive repository of the National Center for Biotechnology Information (NCBI). Accession numbers can be found in Supplementary Table 1. Isolates were collected from 2018 to 2020 and were isolated from nasopharyngeal swabs as described previously (1). Detailed isolate information and characterization can be found in our previous study (1).

Supplementary Table 1: B. pertussis isolates and their origin, SRA accession numbers of newly generated sequencing data.

Isolate	SRA accession no.	Biosample accession no.	Note	Patient location	
**Nanopore raw data**					
Bp-510119	SRR19894764	SAMN29408379		not available	
Bp-510122	SRR19894763	SAMN29408380		not available	
Bp-510285	SRR19894752	SAMN29408381		not available	
Bp-511283	SRR19894741	SAMN29408382		Tyrol, Imst	(1)
Bp-511431	SRR19894730	SAMN29408383		Styria, Leibnitz	(1)
Bp-511432	SRR19894729	SAMN29408384		Styria, Bruck-Mürzzuschlag	(1)
Bp-511495	SRR19894728	SAMN29408385	+ repetition	Upper Austria, Vöcklabruck	(1)
Bp-800127	SRR19894727	SAMN29408386		Salzburg, Sankt Johann im Pongau	(1)
Bp-800128	SRR19894726	SAMN29408387		Tyrol, Innsbruck Land	(1)
Bp-800129	SRR19894725	SAMN29408388		Tyrol, Innsbruck Land	(1)
Bp-800369	SRR19894762	SAMN29408389	+ repetition	Salzburg, Sankt Johann im Pongau	(1)
Bp-800500	SRR19894761	SAMN29408390		Tyrol, Schwaz	(1)
Bp-800629	SRR19894760	SAMN29408391		Salzburg, Salzburg Stadt	(1)
Bp-800630	SRR19894759	SAMN29408392		Salzburg, Salzburg Stadt	(1)
Bp-800631	SRR19894758	SAMN29408393		Salzburg, Salzburg Stadt	(1)
Bp-800941	SRR19894757	SAMN29408394		Salzburg, Salzburg Stadt	(1)
Bp-800944	SRR19894756	SAMN29408395		Salzburg, Salzburg Stadt	(1)
Bp-800945	SRR19894755	SAMN29408396		Salzburg, Salzburg Stadt	(1)
Bp-801006	SRR19894754	SAMN29408397		Salzburg, Salzburg Stadt	(1)
Bp-801761	SRR19894753	SAMN29408398		Salzburg, Sankt Johann im Pongau	(1)
Bp-801838	SRR19894751	SAMN29408399		not available	(1)
Bp-801917	SRR19894750	SAMN29408400	+ repetition	Tyrol, Innsbruck Land	(1)
Bp-802270	SRR19894749	SAMN29408401		Salzburg, Sankt Johann im Pongau	(1)
Bp-802387	SRR19894748	SAMN29408402	+ repetition	Styria, Hartberg-Fürstenfeld	(1)
Bp-802671	SRR19894747	SAMN29408403		Salzburg, Salzburg Stadt	(1)
Bp-803042	SRR19894746	SAMN29408404		Styria, Deutschlandsberg	(1)
Bp-803247	SRR19894745	SAMN29408405		Tyrol, Kufstein	(1)
Bp-804547	SRR19894744	SAMN29408406		Salzburg, Hallein	(1)
Bp-804548	SRR19894743	SAMN29408407		Salzburg, Sankt Johann im Pongau	(1)

Bp-804855	SRR19894742	SAMN29408408		Salzburg, Salzburg Umgebung	(1)
Bp-804857	SRR19894740	SAMN29408409		Salzburg, Salzburg Umgebung	(1)
Bp-804984	SRR19894739	SAMN29408410		Salzburg, Salzburg Stadt	(1)
Bp-806355	SRR19894738	SAMN29408411		Styria, Graz Umgebung	(1)
Bp-806356	SRR19894737	SAMN29408412		Styria, Graz Umgebung	(1)
Bp-806755	SRR19894736	SAMN29408413		Styria, Weiz	(1)
Bp-806901	SRR19894735	SAMN29408414	+ repetition	Styria, Graz Stadt	(1)
Bp-807307	SRR19894734	SAMN29408415		Salzburg, Sankt Johann im Pongau	(1)
Bp-807308	SRR19894733	SAMN29408416		Salzburg, Sankt Johann im Pongau	(1)
Bp-807401	SRR19894732	SAMN29408417		Tyrol, Innsbruck Land	(1)
Bp-807604	SRR19894731	SAMN29408418		Salzburg, Sankt Johann im Pongau	(1)
**Illumina raw data**					
Bp-510119	SRR21002049	SAMN29408379		not available	
Bp-510122	SRR21002048	SAMN29408380		not available	
Bp-510285	SRR21002047	SAMN29408381		not available	
Bp-801838	SRR21002046	SAMN29408399		not available	
Bp-800369	SRR21002055	SAMN29408389	population	Salzburg, Sankt Johann im Pongau	(1)
Bp-800500	SRR21002054	SAMN29408390	population	Tyrol, Schwaz	(1)
Bp-801761	SRR21002053	SAMN29408398	population	Salzburg, Sankt Johann im Pongau	(1)
Bp-801917	SRR21002052	SAMN29408400	population	Tyrol, Innsbruck Land	(1)
Bp-802671	SRR21002051	SAMN29408403	population	Salzburg, Salzburg Stadt	(1)
Bp-806355	SRR21002050	SAMN29408411	population	Styria, Graz Umgebung	(1)
Bp-800631	SRR23314764	SAMN29408393	population	Salzburg, Salzburg Stadt	(1)

Results and Discussion

Evaluation of Nanopore R10.3 flow cells for cgMLST-based analysis of *B. pertussis* isolates

Ten strains were sequenced on an Oxford Nanopore Technologies (ONT) flow cell of the previous generation (10.3) and basecalled in high accuracy mode. A minimum spanning tree based on 2983 cgMLST targets (Figure 1) shows the allelic distance between the assemblies obtained from long read (LR-AS) and gold standard short read assemblies (SR-AS) respectively. Differences in up to six alleles, as for “BP-800128-19”, were observed, which is why that chemistry, flow cell generation and basecalling model were not yet suitable for high-resolution molecular surveillance of *B. pertussis*.

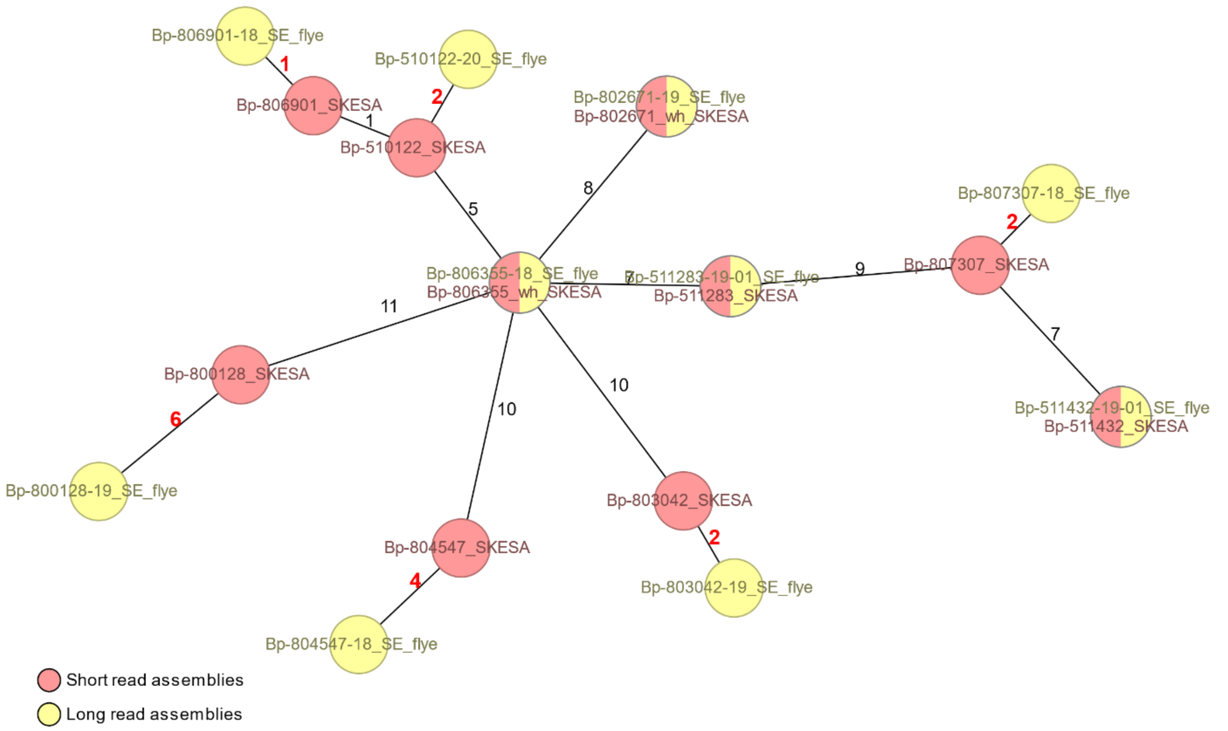

Supplementary Figure 1: Minimum spanning tree of 10 B. pertussis genome assemblies based on short read (red) and long read (yellow) data respectively. Numbers on the lines indicate the differences in the allelic profile between isolates/assemblies.

ONT’s new Q20+ chemistry and flow cells

The recent advances in ONT’s chemistry and flow cell come with an increase in raw read accuracy, as can be seen in Supplementary Figure 2 comparing raw read quality plots from exemplary runs on R10.3 and R10.4 flow cells respectively.

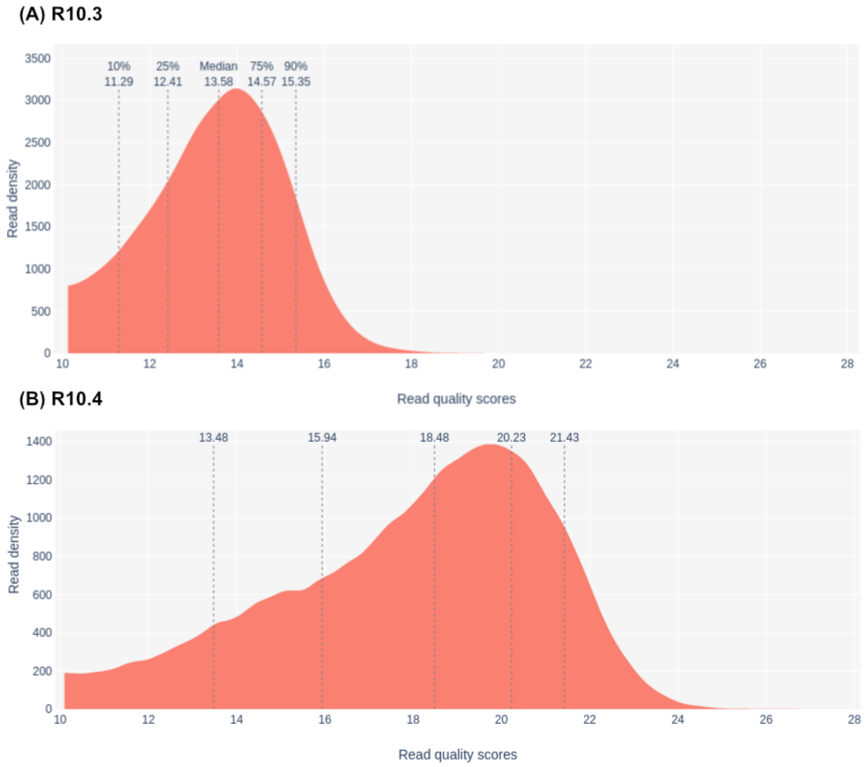

Supplementary Figure 2: Distribution of read quality score plots for an exemplary run on a (A) R10.3 and (B) R10.4 flow cell created by pycoQC (2) show the improved raw read quality of recent flow cell generations.

Establishment of robust pipeline for Q20+ nanopore sequencing and data processing for subsequent cgMLST analysis – detailed comparison and GUI description

The distance matrices show the pairwise genetic distance based on cgMLST targets (Supplementary Table 2) and accessory genome targets (Supplementary Table 3) between assemblies of the different assemblers mentioned in the main text. As can be seen from the pairwise comparison of assemblies of the respective strains, there is not a single difference, except

for one Miniasm assembly. A hybrid assembly was generated by polishing this Miniasm assembly with short reads, which confirmed that the allele identified in the initial Miniasm assembly was wrong.

Supplementary Table 2: Core genome MLST distance matrix of the assembler comparison. Due to size provided as separate excel file.

Supplementary Table 3: Accessory genome MLST Distance matrix of the assembler comparison. Due to size provided as separate excel file.

The number of missing cgMLST targets was tested for significant differences between the assemblers, results are summarized in Supplementary Table 4.

Supplementary Table 4: Statistical comparison of missing cgMLST targets using different assemblers.

Wilcoxon signed rank test (p values adjusted by Holm)	HL esti-mate	95% CI	Below threshold?	Summary	Adjusted P Value	
SR – skesa vs. LR + SR	165.5	148.0 to 174.0	Yes	*	< 0.001	A-B
SR – skesa vs. LR – raven	162.5	146.0 to 173.0	Yes	*	< 0.001	A-C
SR – skesa vs. LR – miniasm	161.5	143.0 to 172.0	Yes	*	< 0.001	A-D
SR – skesa vs. LR – flye	165.5	151.0 to 173.0	Yes	*	< 0.001	A-E
SR – skesa vs. LR – canu	-193.5	-285.0 to -84.0	Yes	*	0.011	A-F
LR + SR vs. LR – raven	-1.5	-7.0 to 0.0	No	ns	0.134	B-C
LR + SR vs. LR – miniasm	-1.5	-5.0 to 0.0	No	ns	0.214	B-D
LR + SR vs. LR – flye	-0.5	-2.0 to 1.0	No	ns	0.516	B-E
LR + SR vs. LR – canu	-358.0	-432.0 to -252.0	Yes	*	< 0.001	B-F
LR – raven vs. LR – miniasm	0.5	-4.0 to 5.0	No	ns	0.965	C-D
LR – raven vs. LR – flye	0.5	-1.0 to 6.0	No	ns	0.415	C-E
LR – raven vs. LR – canu	-353.0	-430.0 to -250.0	Yes	*	< 0.001	C-F
LR – miniasm vs. LR – flye	0.5	-1.0 to 5.0	No	ns	0.516	D-E
LR – miniasm vs. LR – canu	-342.5	-430.0 to -252.0	Yes	*	< 0.001	D-F
LR – flye vs. LR – canu	-357.5	-431.0 to -255.0	Yes	*	< 0.001	E-F

B. NanoAmP Graphical User Interface

We implemented a user interface to facilitate genome assembly based on nanopore data and subsequent typing which can be used for any bacterial species. Using our GUI (see Supplementary Figure 3) the user only has to choose the folder containing the raw reads, select the assembler (Flye, Miniasm/Minipolish, Raven), polishing tools (Racon and/or Medaka) and define the genome size and desired coverage. The last two options are used for filtering the reads with filtlong. For pipeline optimization in the early stages when working with a novel pathogen the user can choose to execute all three assemblers at once to assess the impact of the assembler on cgMLST typing. Since our analysis suggests that a coverage > 100x brings no advantages, as there are anyway no differences to the gold standard, the assembly can be accelerated by downsampling of the data to this value. The implementation of Filtlong ensures in this case however that not random reads but the worst ones are discarded.

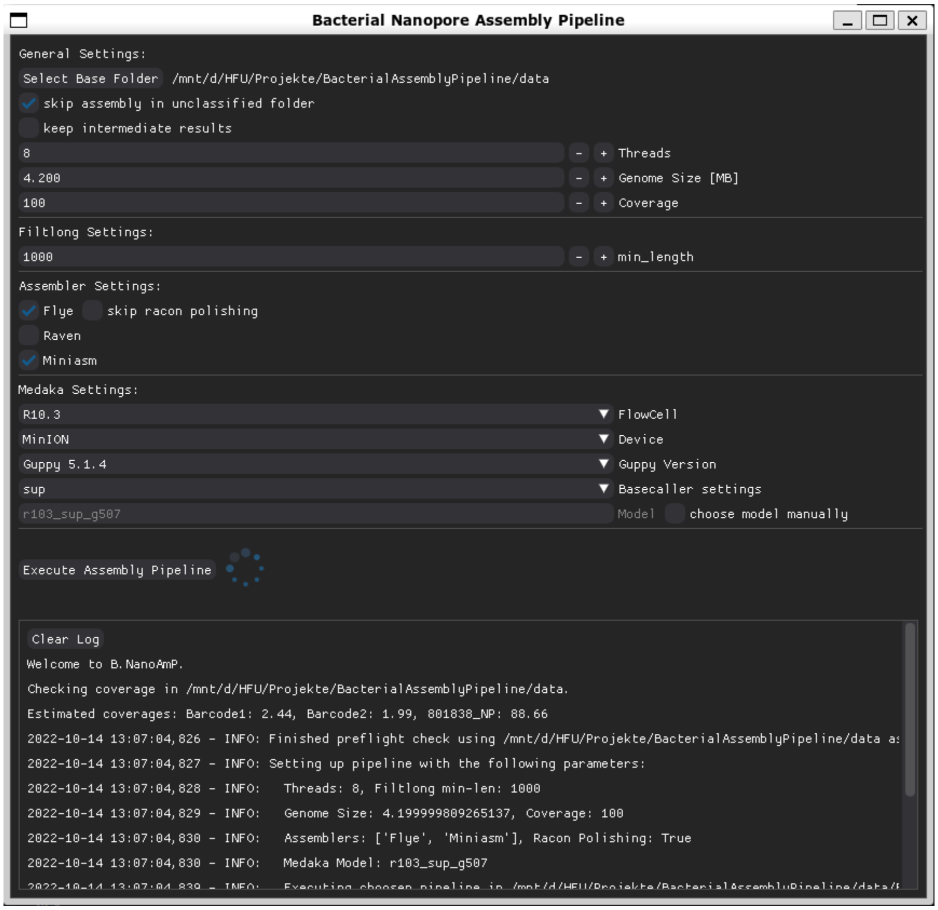

Supplementary Figure 3: Main view of B.NanoAmP. The upper part contains the modifiable parameters and the lower part shows a log with detailed information about the current status and progression of the pipeline.

Low threshold nanopore sequencing catches up with gold standard short-read technologies for high-resolution bacterial pathogen typing – extended analysis

The minimum spanning tree (MST) of hybrid assemblies (HYB-AS) in Supplementary Figure 4 provides the precise genetic distances between isolates. It reconfirms the distances in the MST of LR-AS in the main text (Figure 2), except for a single deviation. Noteworthy, there is one distance between isolates that is larger by one in the SR-AS MST compared to the one of HYB-AS and LR-AS. This is indeed based on a correctly assigned, additional target, which was missing in the LR-AS.

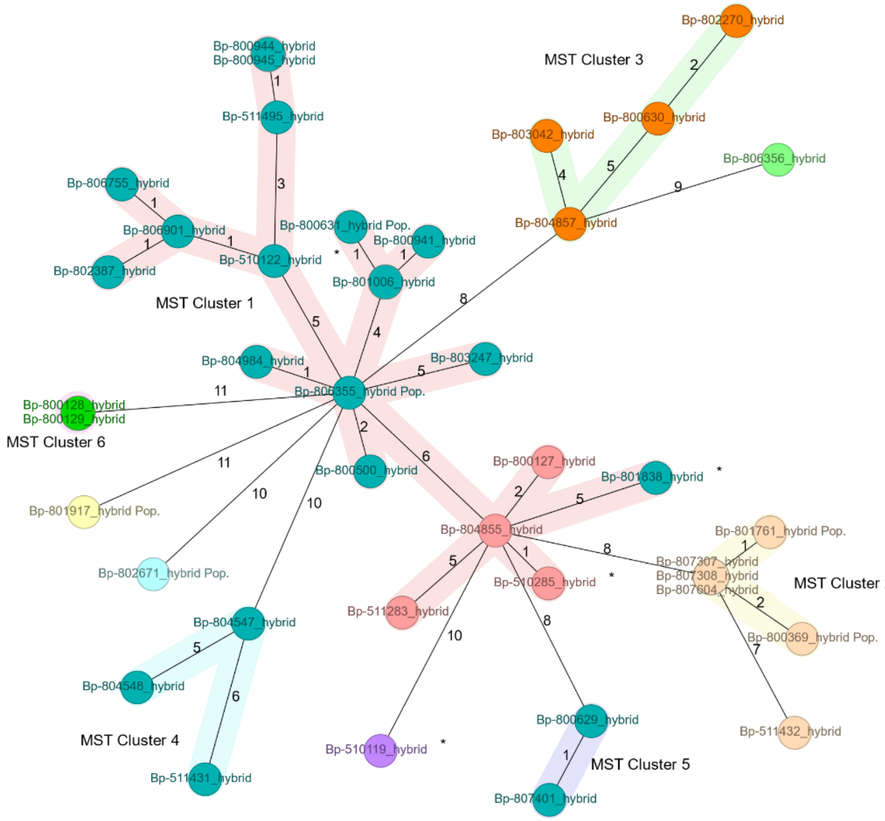

*Supplementary Figure 4: cgMLST-based minimum spanning tree of HYB-AS, comprising the same 40 B. pertussis strains, as the MSTs in the main text. Numbers on lines indicate the number of allelic differences between the respective strains. Cluster threshold was set to six. New strains marked by *, bacterial populations contain "pop". Nodes/strains are colored by genetic profile as denoted in the main text.*

Additional information on base ambiguities frequencies of the population samples for both sequencing technologies can be found in Supplementary Table 5. Reads were mapped (LR with

minimap2 v2.24 (3), SR with BWA v0.7.17 (4)) to reference allele of the denoted locus and base frequencies were obtained by Samtools v1.16.1 (5) and iVar v1.3.1 (6).

Supplementary Table 5: Alternative base frequencies for bacterial population samples of B. pertussis provided for short read (SR-AS) and long read (LR-AS) data.

Strain	Assem-bly	Locus	Posi-tion	Reference base	Alternative Base	Alternative base frequency
Bp-800631	LR-AS	BP0985	668	C	+GGCG	0,25
Bp-800631	SR-AS	BP0985	668	C	+GGCG	0,51
Bp-800369	LR-AS	BP3223	841	T	C	0,56
Bp-800369	SR-AS	BP3223	841	T	C	0,53
Bp-801761	LR-AS	BP0986	719	T	G	0,90
Bp-801761	SR-AS	BP0986	719	T	G	0,88
Bp-801917	LR-AS	BP3554	405	G	A	0,59
Bp-801917	SR-AS	BP3554	405	G	A	0,67
Bp-802671	LR-AS	BP0986	466	G	+CCA	0,54
Bp-802671	SR-AS	BP0986	466	G	+CCA	0,47
Bp-806355	LR-AS	BP0986	719	T	G	0,28
Bp-806355	SR-AS	BP0986	719	T	G	0,00
Bp-800500	LR-AS	BP1877	1997	T	C	0,49
Bp-800500	SR-AS	BP1877	1997	T	C	0,28

As mentioned in the main text, the identified allele variants were identical for all assemblies of the respective strain, SR-AS and LR-AS alike, except for one population sample. This can be easily seen from the diagonal of the distance matrix of all strains and the respective assemblies (see Supplementary Table 6 for the core genome and Supplementary Table 7 for the accessory genome respectively).

Supplementary Table 6: Core genome MLST distance matrix and missing targets of all LR-, SR- and HYB-AS. Due to size provided as separate excel file.

Supplementary Table 7: Accessory genome MLST Distance matrix of all LR-, SR- and HYB-AS. Due to size provided as separate excel file.

Supplementary Table 8 summarizes all the observed differences between LR-AS, SR-AS and HYB-AS on locus level, again showing that these differences are a result from missing loci or an actual ambiguity rather than sequencing errors.

*Supplementary Table 8: Every single deviation in the distance between LR-AS and SR-AS MSTs. The higher distances can mostly be traced back to not found "-" targets. Hybrid assemblies (HYB) show that the identified additional targets in LR-AS are correctly identified and are not due to sequencing errors. The only other difference between SR and LR (in strain Bp-800631) is due to a real base ambiguity in a B. pertussis population sample that was differently resolved in the consensus assemblies. * Indeed an ambiguous base, both variants (1 and 4) are present in locus BP0985 in strain Bp-800631.*

					Allele Strain 1			Allele Strain 2		
**Nr.**	**Strain 1**	**Strain 2**	**Distance**	**Locus**	**LR**	**HYB**	**SR**	**LR**	**HYB**	**SR**
1	Bp-800631	Bp-801006	0 → 1	BP0985	4*	4*	1*	1	1	1
2	Bp-804857	Bp-806356	8 → 9	BP0986	-	1	1	3	3	3
3	Bp-804857	Bp-800630	4 → 5	BP2910	1	1	-	6	6	-
4	Bp-806355	Bp-800500	1 → 2	BP2019	1	1	-	2	2	-
5	Bp-806355	Bp-801917	9 → 11	BP1631	1	1	-	2	2	-
6	Bp-806355	Bp-801917	9 → 11	BP3554	1	1	1	3	3	-
7	Bp-806355	Bp-802671	8 → 10	BP0986	1	1	1	6	6	-
8	Bp-806355	Bp-802671	8 → 10	BP2259	1	1	1	2	2	-
9	Bp-804547	Bp-804548	4 → 5	BP3476	1	1	1	2	2	-
10	Bp-807307	Bp-800369	1 → 2	BP3223	1	1	1	4	4	-
11	Bp-807308	Bp-800369	1 → 2	BP3223	1	1	1	4	4	-
12	Bp-807604	Bp-800369	1 → 2	BP3223	1	1	1	4	4	-

Our LR data typically result in assemblies of a single contig (except for a single LR-AS with two) and hence they are considerably less fragmented than our SR-AS (350.7 contigs on average). Compared to SR-AS, where in the case of our isolates an average of 5.4% (~161.6 loci) of the core genome loci were missing, this value was reduced to 0.3% (~9.1 loci) in the case of LR-AS or hybrid assemblies (~8.6 loci), what fits to our previous results (main text Table 2).

Identification of virulence associated genes and vaccine antigens was achieved using the online BIGSdb-Pasteur Bordetella database and resulted in almost exact same allele assignments between LR- and HYB-AS. A detailed comparison of the results obtained for all assemblies screening several predefined schemes can be found in Supplementary Table 9.

Supplementary Table 9: Typing results for long read assemblies (LR), short read assemblies (SR) and hybrid assemblies (hybrid) of 40 B. pertussis isolates each screening the BIGSdb-Pasteur Bordetella database (schemes: “Bp_vaccine antigens”, “macrolide resistance”, “Autotransporters”, “T3SS”, “Phase” and “Other toxins”)[22,55]. Missing targets marked with “x” and yellow fill color. Red fill color indicates differences in the identified alleles depending on the sequencing technology used. Due to size provided as separate excel file.

1. Cabal A, Schmid D, Hell M, Chakeri A, Mustafa-Korninger E, Wojna A, Stoger A, Most J, Leitner E, Hyden P, Rattei T, Habington A, Wiedermann U, Allerberger F, Ruppitsch W. 2021. Isolate-Based Surveillance of Bordetella pertussis, Austria, 2018-2020. Emerg Infect Dis 27:862-871.
2. Leger A, Leonardi T. 2019. pycoQC, interactive quality control for Oxford Nanopore Sequencing. Journal of Open Source Software 4.
3. Li H. 2021. New strategies to improve minimap2 alignment accuracy. Bioinformatics doi:10.1093/bioinformatics/btab705.
4. Li H. 2013. Aligning sequence reads, clone sequences and assembly contigs with BWA-MEM. arXiv preprint arXiv:13033997.
5. Danecek P, Bonfield JK, Liddle J, Marshall J, Ohan V, Pollard MO, Whitwham A, Keane T, McCarthy SA, Davies RM, Li H. 2021. Twelve years of SAMtools and BCFtools. Gigascience 10.
6. Grubaugh ND, Gangavarapu K, Quick J, Matteson NL, De Jesus JG, Main BJ, Tan AL, Paul LM, Brackney DE, Grewal S, Gurfield N, Van Rompay KKA, Isern S, Michael SF, Coffey LL, Loman NJ, Andersen KG. 2019. An amplicon-based sequencing framework for accurately measuring intrahost virus diversity using PrimalSeq and iVar. Genome Biology 20:8.
